# Spontaneous Late Intraocular Lens and Capsule Tension Ring Dislocation

**DOI:** 10.4274/tjo.79836

**Published:** 2017-04-01

**Authors:** Ayşe Gül Koçak Altıntaş, Aslıhan Esra Omay, Selda Çelik

**Affiliations:** 1 Ulucanlar Eye Training and Research Hospital, Ophthalmology Clinic, Ankara, Turkey

**Keywords:** Capsule contraction syndrome, capsule tension ring, intraocular lens dislocation, pseudoexfoliation

## Abstract

In this report, three cases with pseudoexfoliation (PEX) and advanced age with spontaneous intraocular lens (IOL) and capsule tension ring (CTR) dislocation were presented. All of our cases experienced progressive vision loss without an episode of strenuous physical activity, trauma, or any other ocular disease. Spontaneous dislocation was observed 2.5 to 8 years after uneventful phacosurgery. Each patient underwent complete IOL and CTR removal combined with anterior chamber IOL implantation. No complications were noticed during follow-up. As a result, capsule tension ring does not prevent late IOL dislocation after uncomplicated phacosurgery in the presence of PEX. Therefore, close follow-up is essential for patients with PEX.

## INTRODUCTION

Capsule contraction syndrome (CCS) is myofibroblastic metaplasia of the anterior lens epithelia cells (LECs) and exaggerated contraction of both fibrotic anterior capsulectomy opening and capsular bag diameter.^1,2^ These changes could lead to intraocular lens (IOL) decentration within the capsular bag followed by dislocation or total IOL luxation into the vitreous cavity.^3,4,5^ Early IOL dislocation occurs in cataract surgery with inadequate support for the IOL resulting from intraoperative zonular or capsular damage, but late dislocation following uncomplicated surgery usually occurs several months to many years postoperatively.^4,5,6,7,8^

Several ocular and systemic factors for CCS and IOL dislocation have been identified, such as pseudoexfoliation syndrome (PEX), advanced age, trauma, high myopia, diabetes mellitus, uveitis, certain connective tissue disorders, and previous vitreoretinal surgery.^2,9,10,11,12^ Surgical factors including capsulorhexis size and IOL design and materials may also influence the development of CCS and IOL dislocation.^4,5,12^

Here, we present three cases with PEX who developed subluxation of IOL and capsular tension ring (CTR) combined with fibrotic capsular bag, after uneventful phacoemulsification.

## CASE REPORTS

### Case 1

**Figure 1:**
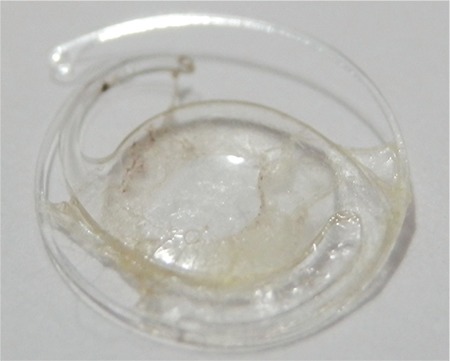
Intraocular lens and capsular tension ring in the fibrotic capsular bag

A 72-year-old man presented with symptoms of marked reduction of vision 3 years after cataract surgery on his right eye. He had undergone cataract extraction with anterior chamber IOL implantation in his left eye at another hospital five years before the phacoemulsification surgery on his right eye. Bilateral PEX and phacodonesis in his right eye were noted in his preoperative medical records from our hospital. Therefore, during the uneventful phacoemulsification, one-piece hydrophilic acrylic foldable IOL and CTR implantation was performed. The patient was followed for 3 months, and his visual acuity (VA) was 6/10 and no complication was observed at his final examination. Three years after his uneventful right eye operation, the patient presented to our clinic with complaint of blurred vision early in the morning upon waking. Slit-lamp examination revealed subluxation of the IOL and CTR inside the fibrotic and contracted capsular bag. The subluxation was inferior and towards the posterior when the patient was in supine position. Intraocular pressures (IOP) were 14 and 16 mmHg for right eye and left eye respectively. Fundus examination revealed bilateral dry age-related macular degeneration. The IOL and CTR in the fibrotic capsular bag were extracted with IOL forceps in order to prevent luxation into the vitreous (Figure 1). After performing anterior vitrectomy and inducing miosis with intracameral 0.5 mL of 0.01% carbachol solution (Miostat^®^, Alcon, USA), a one-piece polymethyl methacrylate (PMMA) IOL was implanted into the anterior chamber. At final examination at postoperative 6 months, the patient’s VA in the right eye had improved to 6/10 and slit-lamp examination revealed the IOL was stable, in correct position with normal pupil shape and without any anterior chamber reaction.

### Case 2

**Figure 2:**
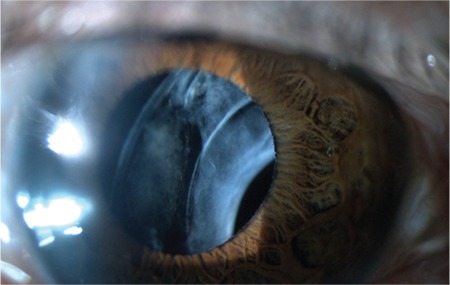
Subluxated in-the-bag intraocular lens and capsular tension ring

A 76-year-old man was admitted to our clinic with complaint of left-sided visual deterioration. He had undergone phacoemulsification in the left eye two and a half years earlier. His VA was counting fingers from 2 meters and did not improve with correction. On slit-lamp examination of his left eye, pseudoexfoliation material and significant inferior dislocation of the IOL and CTR was observed, and anterior capsule phimosis with a marked decrease in capsular bag diameter was noted (Figure 2). Examination of his right eye revealed marked pseudophacodonesis, nuclear sclerosis, and pseudoexfoliation material at the pupillary margins. Dry age-related macular degenerations were observed. According to his medical records, CTR implantation was done for prophylactic support after a two clock-hour area of zonular dehiscence was recognized intraoperatively. However, no other intraoperative or postoperative complications had been recorded during the regular three-month follow-up period. Because of significant inferior subluxation, complete capsular bag extraction with IOL and CTR followed by anterior vitrectomy combined with anterior chamber IOL implantation were performed to prevent total drop of IOL and CTR into the vitreous, similar to case 1. The patient’s best corrected VA improved to 5/10 postoperatively.

### Case 3

**Figure 3:**
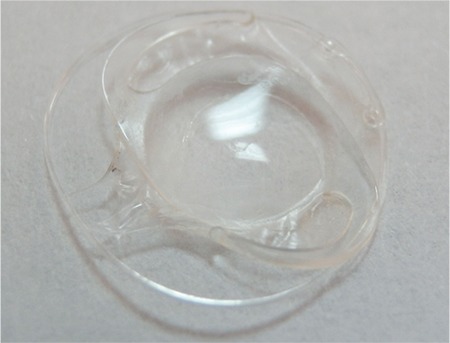
Intraocular lens and capsular tension ring folding in the fibrotic capsular bag

A 79-year-old woman reported progressive visual blurring in the left eye for nearly 6 months. Her VA was counting fingers from 1 meter in the left eye and 20/20 in the fellow eye. According to her medical records, she had undergone uneventful phacoemulsification and one-piece foldable hydrophilic acrylic IOL implantation surgery in both eyes 8 years earlier. She was being medically treated for glaucoma in the right eye and had previously undergone trabeculectomy surgery on her left eye. Despite the absence of significant zonular weakness, CTRs had been implanted in both eyes for preventive purposes due to substantial accumulations of PEX material on both the pupillary margins and anterior capsular surfaces. Slit-lamp examination of the right eye showed bilateral pseudoexfoliation at the pupillary margin and stable IOL in the bag, whereas the left eye showed pronounced inferior dislocation of the IOL and CTR and total curving of the CTR inside the fibrotic and significantly constricted capsular bag. The patient underwent surgery to remove the subluxated capsular bag containing the IOL and CTR followed by anterior chamber IOL implantation (Figure 3). Her VA in the left eye was 6/10 at postoperative 1 month and there were no complications. Fundoscopic evaluation revealed cup-to-disc ratios of 6/10 and 8/10 and IOPs of 14 and 16 mmHg in the right eye and left eye, respectively.

Each of these three cases denied trauma, any other ocular surgery after cataract extraction or ocular disease other than PEX, glaucoma and age-related macular degeneration.

## DISCUSSION

IOL dislocation or decentration is a rare but serious complication following uneventful phacoemulsification.^4^ The incidence of repositioning or exchange procedures for dislocated IOL ranges between 0.2% and 3%.^5,13^ Mönestam^5^ reported that 0.6% of patients were at risk of required surgery for a dislocated IOL 10 years after initial surgery.

Cuboidal anterior lens epithelial cell metaplasia and myofibroblastic transformation to actin-positive smooth muscle cause extreme shrinkage of the capsular bag. This progressive contraction can directly result in zonular dehiscence, leading to IOL and capsular complex dislocation.^11,14,15^ Fibrotic contraction and opacification of the anterior capsule usually occurs 3 to 6 months after surgery, but spontaneous dislocation of the IOL with CCS and extensive constriction of the capsular bag following uncomplicated surgery may occur many months to several years later.^4,6^ Kumar et al.^4^ reported two cases aged 83 and 74 years with IOL dislocation within the bag 3 and 6 months after surgery, respectively. To correct visual deterioration they had removed dislocated IOLs from the bag and implanted rigid IOLs in the sulcus for each patient. Coelho et al.^3^ reported a 58-year-old patient with significant inferonasal subluxation of the IOL with a contracted capsular bag 3 years after surgery, similar to our cases. To prevent luxation into the vitreous, their patient underwent complete capsular bag and IOL removal and implantation of another IOL in the ciliary sulcus by scleral fixation. Suturing the IOL and CTR combined with capsular bag into the sclera may have potential complications such as retinal tearing, retinal detachment, and vitreous hemorrhage. Due to these potential complications and the presence of significant capsular folding in our patients, we did not prefer scleral fixation.

We observed dislocation 2.5 to 8 years after uneventful phacosurgery, similar to Coelho et al.’s^3^ case. These observations support that dislocations may occur much later than CCS development. Consistent with that case, we preferred extraction of the subluxated IOL and CTR with fibrotic capsular bag in our cases. However, our cases were much older than Coelho et al.’s^3^ patient, and we therefore decided to implant the IOL into the anterior chamber. None of our patients had any complications associated with the anterior chamber IOL like corneal edema or anterior chamber flare or cells.

If a recent ocular trauma is ruled out, several predisposing factors such as pseudoexfoliation, advanced age, diabetes mellitus, and high myopia may lead to zonular dehiscence.^3^ We observed PEX in each of our three cases and they all had advanced age as risk factor for dislocation.^5^ Mönestam^5^ reported PEX in 4 out of 5 patients with IOL dislocation. In a comparative study, Hayashi et al.^9^ noted greater IOL tilt in eyes with PEX than in otherwise healthy eyes.

A CTR improves capsular stability and prevents focal stress on compromised zonules. CTR implantations were performed to maintain equally distributed equatorial forces and circular contour of the capsular bag to provide zonular stability due to the presence of preoperative phacodonesis in case 1 and intraoperative focal dehiscence in case 2. In cases 1 and 2, IOL and CTR dislocation were noticed earlier than case 3, in whom CTR insertion was done even in the absence of any risk factors other than PEX. Case 3 developed IOL and CTR dislocation 8 years after surgery; to our knowledge, this is the latest spontaneous dislocation reported in the literature.

Different clinical and historical studies have demonstrated that zonules were fragile and had weak stretching capability in PEX.^9,16^ Severe capsular fibrosis causes an imbalance between centrifugal and centripetal forces on the capsular bag, which may cause progressive dehiscence of the zonules and spontaneous in-the-bag IOL dislocation. The use of CTRs may reduce the risk of zonular rupture but does not guarantee zonular stability or prevention of spontaneous late IOL dislocation combined with fibrotic capsular bag, as we observed in all three our cases, even in the absence of any preoperative or postoperative complications other than PEX and advanced age.^5,17,18,19,20,21,22^ Our patients had experienced progressive vision loss without any precipitating strenuous physical activity, trauma or ocular disease. It is reported that CCS is influenced by IOL design, both the optic and haptic material, and more specifically the hydrophilicity of the optic biomaterial. However, the occurrence of CCS with silicone, PMMA, and both hydrophilic and hydrophobic acrylic IOLs has been reported, despite the use of CTRs.^1,23,24,25,26^

## CONCLUSION

According to our observations, the use of CTR regardless of optic and haptic material cannot always resist the centripetal forces generated by fibrotic contracting capsulorhexis. There are no specific measures to prevent late postoperative subluxation, but avoiding small capsulorexis, carefully cleaning the posterior capsule and completely removing all cortical material, especially in the equatorial area, are important steps to prevent CCS. Selecting a hydrophobic IOL with a sharp optical posterior edge and inserting a CTR in suspected cases can also reduce the risk of late CCS development. Despite all of these measures, late CCS is unavoidable in some cases, especially eyes with PEX. Therefore, CTR with scleral fixation may prevent late CCS in cases with high risk, such as eyes with PEX and defective zonules. Furthermore, prolonging close follow-up as long as possible is essential for early diagnosis and prompt treatment of patients at risk of spontaneous IOL decentration or subluxation.
